# A large uterine leiomyoma leading to non-puerperal uterine inversion: A case report

**Published:** 2017-01

**Authors:** Batool Teimoori, Arezoo Esmailzadeh

**Affiliations:** *Department of Obstetrics and Gynecology, Zahedan University of Medical Sciences, Zahedan, Iran.*

**Keywords:** Leiomyoma, Uterine inversion, Haultain’s procedure, Laparotomy

## Abstract

**Background:**

Although leiomyomas are the most common gynecologic disorders, non-puerperal uterine inversion due to leiomyoma is considered as a rare clinical problem. This condition can occur as a complication of a large sub-mucous leiomyoma that leads to dilate cervix and protrude into vagina. The patient may have several symptoms such as heavy vaginal bleeding, pelvic pain and intermittent acute urinary retention.

**Case:**

We presented a 32-year-old nulliparous woman with 17 years of unexplained infertility and diagnosis of a large vaginal prolapsed non-pedunculated leiomyoma.

**Conclusion:**

Haultain’s procedure was used to reposition uterine inversion and remove leiomyoma through a posterior incision, using laparotomy.

## Introduction

Uterine inversions are classified into two groups, including (a) puerperal and (b) non-puerperal inversions. Most cases have reported puerperal uterine inversion as a rare condition (1). Leiomyomas are considered as the most frequent gynecologic problems. A report has been shown that submucosal leiomyoma dilated cervix and prolapsed into the vagina (2). Furthermore, some cases of prolapsed cervical myoma may show some malignancy features that lead to differential diagnoses, and frozen section analysis during surgery is considered as a logical action (3). 

Non-puerperal uterine inversion caused by submucousal leiomyoma has been more frequently reported in African women (4). The clinical diagnosis of non-puerperal uterine inversion is divided into (a) chronic signs including irregular vaginal bleeding, anemia, and a feeling of mass coming down in vagina and (b) acute signs including pelvic pain and heavy vaginal bleeding (5). Some literatures have also pointed to vaginal discharge, urethrovaginal fistula and intermittent acute urinary retention (3). 

Types of treatment vary greatly from case to case. Abdominal and vaginal hysterectomies are recommended for women who have completed their family size. Vaginal myomectomy is suggested for the cases showing no malignancy. Finally, for uterine inversion caused by malignancy, advanced surgery such as radical abdominal hysterectomy is indicated. We used Haultain’s procedure to reposition uterine inversion after removing leiomyoma through a posterior incision in a case of a 32-year-old nulliparous woman with a 17-year history of infertility and diagnosis of a large vaginal prolapsed non-pedunculated leiomyoma. 

## Case report

A 32-year-old nulliparous woman with a 17-year history of unexplained infertility and previous spontaneous miscarriage presented to Ali-Ebne Abi-Taleb Hospital, Zahedan, Iran, in January 2016. The first manifestation was a temporary loss of consciousness when presenting to the hospital. Clinical examination showed an ill-looking woman with temperature of 37.2^o^C, pulse rate of 112 bpm and blood pressure of 90/60 mmHg. 

A pelvic exam showed a non-pedunculated mass coming into vagina about 2 cm above hymen that was closed vaginal channel, whereas she experienced no feeling of mass coming down in the vagina. She had a recent history of amenorrhea, postcoital bleeding, and cloudy-white/transparent vaginal discharge. She never pursued infertility treatment and never had a pap test. The results of blood test showed hemoglobin of 11.2 gm/dL and normal levels of blood urea nitrogen (BUN), creatinine (Cr), sodium (Na), and potassium (K), and the result of urinalysis (UA) was also normal. Since her abdominal ultrasound showed no useful results, she was prepared for genital examination under general anesthesia. The findings showed that a mass filled the whole vaginal tract, and the cervix could not be differentiated. After starting laparotomy forcefully, Haultain’s procedure was used to reposition uterine inversion and remove leiomyoma through a posterior incision. 

The size and weight of leiomyoma excised were 77×65 mm and about 413 gr without stalk, respectively. She was discharged from the hospital on the third postoperative day in a good clinical condition.

**Figure 1:**
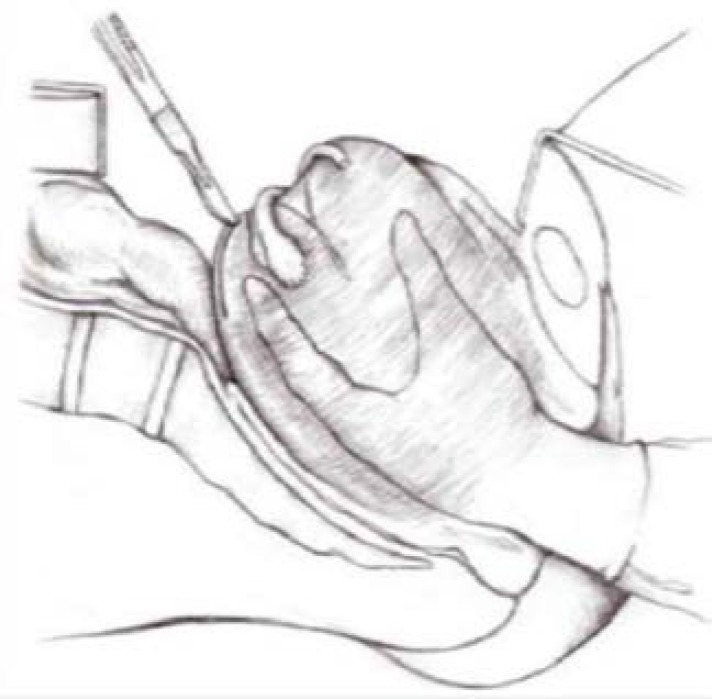
Haultain’s procedure

## Discussion

Uterine inversions are classified into two groups, including (a) puerperal that is due to obstetric problem and (b) non-puerperal inversions that is due to gynecological problem (6). Vaginal myomectomy is considered as a common and effective treatment, but in our case of uterine inversion due to submucosal leiomyoma, leiomyoma did not connect to uterus through a stalk (2). Vaginal myomectomy was not considered as an option. 

Due to presence of large mass in vaginal tract, Haultain’s procedure was used to reposition uterine inversion and remove leiomyoma through a posterior incision, using laparotomy forcefully. In several studies, vaginal myomectomy followed by vaginal hysterectomy has been considered. This method is mainly recommended for women who have completed their family size, but our patient had no child due to unexplained infertility. Following laparotomy, she may have a chance of getting pregnant in future after treating her infertility. 

Furthermore, in a follow-up, her pathology result was benign. We can nevertheless confirm that there are some limitations in our study. Firstly, we did not use transrectal ultrasonography (TRUS). Secondly, our patient desired to return to the physician for treating her infertility. 

## Conflict of interest

The authors declared no conflict of interest.
